# Electives in the medical curriculum – an opportunity to achieve students’ satisfaction?

**DOI:** 10.1186/s12909-020-02269-0

**Published:** 2020-11-23

**Authors:** Ana Rita Ramalho, P. M. Vieira-Marques, C. Magalhães-Alves, M. Severo, M. A. Ferreira, I. Falcão-Pires

**Affiliations:** 1 Department of Public Health and Forensic Sciences and Medical Education, Faculty of Medicine of University of Porto, Porto, Portugal; 2 CINTESIS - Center for Research in Health Technologies and Information Systems. Faculty of Medicine, University of Porto, Porto, Portugal; 3 Cardiovascular Research and Development Center, Faculty of Medicine of the University of Porto, Porto, Portugal

**Keywords:** Undergraduate medical curriculum, Elective, Alternative curricula, Teaching and learning, Workload, Assessment, Satisfaction

## Abstract

**Background:**

Electives are perceived by medical students as a valuable, highly regarded experience, allowing them to customize learning experiences and enabling them to early differentiate during medical training. The present work aims to uncover students’ major determinants of satisfaction and how they interfere with their future elective choices in order to identify the best approach to implement electives in medical curricula.

**Methods:**

A cross-sectional study was conducted through a written evaluation survey concerning the electives available in the academic year 2015–2016. Our institution provides 106 electives to students from the 2^nd^ to the 5^th^ year. Students’ satisfaction was assessed through a validated questionnaire with eight sentences expressing opinions related to electives global satisfaction. Data from 538 inquiries from 229 students were analyzed quantitatively using regression and correlation models, and qualitatively through phenomenography.

**Results:**

Quantitative analysis of the questionnaires allowed to establish both: 1) The determinants of students’ satisfaction with electives, which were *Agreement with teaching and learning methodologies,* followed by *Agreement with assessment methodologies employed*, *Perception of the workload demanded* and *Requirement for continuous work* and 2) The predictors of students preferences in the following years, namely, *Agreement with assessment methodologies employed*, *Classes attendance* and *Ranking of the allocated elective established in the previous year*. Qualitative analysis of questionnaires revealed that students consider electives as being innovative and interesting, claiming that some, for their relevant content, could be integrated into the medical core curriculum.

**Conclusions:**

Our work raises awareness on the best practices when it comes to electives’ organization to meet students’ satisfaction. We can conclude that medical schools should measure students satisfaction as a tool to organize and predict future needs of electives and placements when designing and implementing this alternative student-centred curriculum or even to improve the existing practices regarding electives in medical courses.

## Background

With the publication of the Flexner Report in 1910, medical teaching and curriculum progressed towards standardisation [1]. Since then, a change in the paradigm led to the implementation of alternative curricula pathways in addition to the core curriculum, including shortened preclinical curricula, dedicated research time and early clinical experiences [2]. These alternative curricular pathways are designated as “electives” and, although there is no standard definition, the term infers to “a period of time during undergraduate within which there is a significant element of student choice*”* [3]. Students perceive electives as a valuable, highly regarded experience [4], with benefits in providing a better learning and academic success, delivered by the possibility of personalizing medical curricula and stimulating students’ self-motivation [5].

Basic science and clinical syllabus remain the strongest foundation of medical curricula. Nevertheless, electives emerge as a complement to this core curriculum as they provide students with the opportunity to customise their studies by selecting, directing and organising the curricula that best meets their individual needs and/or interests [3, 6]. Furthermore, electives may represent the recognition by medical schools of new emerging scientific areas, the accomplishment of the growing importance of the biomedical, statistical, social and epidemiologic sciences as well as information technologies, and the need for a novel strategy to cope with the constraints of an expanding body of knowledge while having a limited amount of educational time [2].

Therefore, the inclusion of electives in medical degree curricula is of growing importance. For accreditation of medical education, Standard 6 from the Liason Committee on Medical Education describes the competencies, curricular objectives and curricular design [7]. This standard mentions that medical curriculum should “include elective opportunities that supplement required learning experiences and that permit medical students to gain exposure to and deepen their understanding of medical specialties reflecting their career interests and to pursue their individual academic interests”. Notwithstanding their emerging relevance in medical curricula, literature about electives is scarce, probably being the least researched component of undergraduate medical education [8]. The existing work focuses on electives that are initiated and organized entirely by students, as well as on the benefits of free choice clerkship electives in medical education, including its relevance for students’ future education and career choices. While, for some students, electives reaffirm their will to pursue a specific career, for others, they provide insight that will ultimately facilitate future career choices [4]. However, there seems to have been only a narrative exposition of this occurrence [4], such as the consensus statement published by the Medical Schools Council Electives Committee that compiled and reported such experiences, after acknowledging the limited amount of literature about design and management undergraduate elective curricula [9].

Currently, some authors have assessed the functioning of electives based on formative and summative feedback, enabling individual conclusions for each elective’s future [10]. Additionally, a study conducted by Maki and Maki predicted the variables that interfered with satisfaction in different typologies of courses [11]. However, information regarding the electives’ consistent evaluation, its global impact on course functioning, as well as the influence of the variables in the context of electives are still missing. Thus, the first goal of this work is to assess students’ perceptions of the opportunities that electives create in medical curricula. The second goal is to provide insights about the best approach to implement electives in medical curricula regarding its organization, methodologies of assessment and classes typology in order to meet students' satisfaction. Lastly, we aim to assess predictor factors derived from students’ satisfaction with electives that might influence their engagement, satisfaction and elective choices in the following years, indirectly allowing for better management of resources allocation.

## Methods

### Electives in Faculty of Medicine of University of Porto (FMUP)

FMUP provides 106 electives (1.5 or 3 ECTS corresponding to 14 or 24 h of total workload) grouped in 4 different scientific areas (biomedical sciences, social and human sciences, clinical sciences and information and technology sciences). In the academic year of 2015/2016, only 40 electives were selected by medical students and, therefore, only 40 electives were assessed.

There are no compulsory electives. The only fixed criteria is that, from the 2^nd^ to the 5^th^ year of medical school, each student must select between 3 and 9 ECTS from each one of these areas to acquire a balanced knowledge and curricula. Students have to complete 24 ECTS out of the 360 ECTS that comprise the medical course of FMUP. The majority of electives are lectured at FMUP’s facilities and students are covered by a pre-graduate insurance during dislocations within University of Porto facilities or to any affiliated hospital.

### The written evaluation survey

A cross-sectional study was conducted, concerning the evaluation of electives available in the academic year of 2015/2016. Consent was obtained from the students and participation was voluntary.

A written evaluation survey (see Additional File 1) was developed to assess students’ satisfaction with electives and to identify the determinants of student’s future choices (Table 1).

**Table 1:** Description and corresponding grade of the variables considered in the written evaluation survey

Variables	Description of the variable and its assessment methodology	Variables grade
I **Gender and Age**	Both students’ gender and age were recorded via self-report.	0: female 1: male
II **Grade**	Students’ grade obtained in the elective was recorded via self-report.	
III **Workload Perception**	Workload is the work entailed to successfully complete the assignments, tasks and examinations. Perception of the elective’s workload was assessed through a questionnaire with five sentences reporting feelings related with the elective amount of work.	Students were asked to choose from 1 point [totally disagree] to 5 points [completely agree], making a total of 25 points. The final score of workload perception was given by the sum of all items. The higher the score obtained, the higher the students’ workload perception [12].
IV **Attendance**	The attendance of the elective was assessed using a retrospective self-attendance frequency questionnaire that included three questions: 4.1) *How often did you attended the classes of the elective?* 4.2) *How often did you study for that elective during the exams period?* 4.3) *On the days that you studied, how many hours did you study on average per day?*.	For the first two questions, students had a Likert scale of response options to choose from. For the first question: 0%, 1 to 24%, 25 to 49%, 50 to 74%, 75 to 99 and 100%. For the second question: never; once or less than once a month; 2 or 3 times per month; once a week, twice a week, 3 to 4 times per week; 4 times per week; and 6 to 7 times per week. For the third question: the number of hours was recorded through self-report. To calculate the amount of self-study per week, the selected frequency category was converted to mean times/week and multiplied by the number of self-study hours [13].
V **Assessment in the exams’ period**	Students were asked if the elective had its assessment occurring during exams’ period.	If the answer was affirmative, students were asked to indicate both the number of days spent studying during the time of exams and the number of hours dispended per day studying through self-report.
VI **Global Satisfaction**	Global satisfaction of students with an elective was assessed through a questionnaire with eight sentences reporting feelings related with global satisfaction.	Students were asked to choose from 1 point [totally disagree] to 5 points [totally agree], making a total of 40 points. The final score of students’ global satisfaction was given by the sum of all items. The higher the score obtained, the higher the level of global satisfaction of students with the elective they attended.
VII **Assessment Methodology**	Students were asked if they agreed with both the assessment methodology in the elective and with the weight of each assessment component.	If students did not agree, they were asked to choose from a set of assessment methodologies and to attribute one weight to it, according to their self-perception of which the assessment methodology of the elective they assigned to should be.
VIII **Typology of Classes**	Students were asked if they agreed with the typology of classes applied in the elective.	If students did not agree, they were asked to choose from a set of typologies according to their self-perception of what the typology of classes of the elective they assigned to should be, with the possibility to add a new suggestion of typology.
IX **Estimated Workload**	The estimated workload of the elective was assessed through a NASA-Task Load Index, a multi-dimensional scale proven to be reliable in obtaining workload estimates [14], consisting of six subscales that represent independent cluster of variables: mental, physical and temporal demands, performance, effort and frustration.	For each variable, students were asked to choose the answer that best represents their effort in the accomplishment of the elective in a scale from 1 point [low] to 10 points [high]. The final score of estimated workload was given by the simple sum of the individual scales. The higher the score obtained, the higher the estimated workload [15].

The written survey was handed once to students who attended electives when they were in the 2^nd^ and 3^rd^ years, in the academic year of 2015/2016. In order to maximise the number of surveys answered, these were delivered in hand to students during one of the most attended lectures of each curricular year. Whereas each student answered one survey, each survey included as many inquiries as the number of electives attended by the student in the academic year of 2015/2016, counting one to four inquiries.

After some close-ended questions, the inquiry was concluded with an optional open-ended question to collect students’ opinion about the electives they attended in the previous year.

Finally, other variables were retrieved from an information system developed to allocate electives according to students preferences (Table 2).

**Table 2:** Description of the variables gathered from an information system developed to support the allocation procedure of electives

Variables	Description
X. **Number of Students**	The number of students corresponds to the number of students attributed to one elective by an information system developed to support the allocation procedure of electives. This variable was obtained from the same information system.
XI. **Order of Preference**	The order of preference was obtained from the records of an information system developed to support the allocation procedure of electives. The higher the value of the elective in students’ personal order of preference, the lesser the student preferred the elective.

### Statistical analysis

Concerning the survey applied, when studying the correlation between two variables and their ability to determine students’ global satisfaction with an elective, we demonstrated statistically significant correlations between two variables that, when associated, interfered with each other and, consequently, with students’ global satisfaction, which we aimed to assess.

The variables grade and perception of workload were standardized by Z-score transformation. A simple linear regression and a multiple linear regression were used to show the crude and adjusted associations, respectively.

The linear association between every considered variable, corresponding each variable to one possible determinant of students’ global satisfaction with electives, was evaluated. Pearson, Polychoric and Point Bi-serial correlations according to the type of scale used in each variable were employed (Table 3).

**Table 3:** Correlation between several possible determinants of students’ global satisfaction with electives

	Gender	Age **(year)**	Grade	Agrees with assessment methodology	Perception of workload	Attendance	Agrees with teaching methodology	Score NASA	Frequency of study > 0	Number of students in 2015/2016 **(N.x/100)**	Order of preference in 2015/2016 **(V1.x)**
**Gender**	1	Point Bi-serial	Point Bi-serial	Polychoric Correlation	Point Bi-serial	Point Bi-serial	Point Bi-serial	Point Bi-serial	Polychoric Correlation	Point Bi-serial	Point Bi-serial
**Age** (year)	−0.206 *	1	Pearson	Point Bi-serial	Pearson	Pearson	Pearson	Pearson	Point Bi-serial	Pearson	Pearson
**Grade** (0–20)	− 0.128 *	− 0.010 *	1	Point Bi-serial	Pearson	Pearson	Pearson	Pearson	Point Bi-serial	Pearson	Pearson
**Agrees with assessment methodology**	0.027	0.027 *	0.469	1	Point Bi-serial	Point Bi-serial	Point Bi-serial	Point Bi-serial	Polychoric Correlation	Point Bi-serial	Point Bi-serial
**Perception of workload** (5–25)	0.063	0.028 *	−0.407	− 0.499	1	Pearson	Pearson	Pearson	Point Bi-serial	Pearson	Pearson
**Attendance**	−0.159 *	−0.032 *	0.134	−0.030 *	− 0.026 *	1	Pearson	Pearson	Point Bi-serial	Pearson	Pearson
**Agrees with teaching methodology**	0.076	0.016 *	0.132	0.366	−0.146	− 0.067 *	1	Pearson	Point Bi-serial	Pearson	Pearson
**Score NASA** (1–10)	−0.009	−0.025 *	− 0.211	−0.315	0.573	0.064 *	−0.097	1	Point Bi-serial	Pearson	Pearson
**Frequency of study > 0**	−0.113	0.091	−0.133	−0.115 *	0.344	0.076 *	−0.046 *	0.350	1	Point Bi-serial	Point Bi-serial
**Number of students in 2015/2016** (N.x/100)	−0.057	−0.078 *	0.380	0.330	−0.326	0.058 *	0.178	−0.128	−0.244	1	Pearson
**Order of preference in 2015/2016** (V1.x)	0.060	0.063 *	−0.194	−0.033 *	0.215	0.084 *	−0.003 *	0.172	0.153	−0.274	1

* *p* < 0.05

For the outcome of evaluating the determinants on students’ global satisfaction with electives (Table 4), the crude and adjusted regression coefficients for every variable were shown. Simple linear regression and a multiple linear regression were used to show the crude and adjusted association, respectively.

**Table 4:** Evaluation of the determinants of students’ global satisfaction with electives

	ß crude	(95% CI)	ß adjusted	(95% CI)
Gender				
Female	Ref			
Male	−0.503	(−1.968;0.962)	0.135	(−0.899;1.168)
Age (year)	0.017	(−0.250; 0.284)	0.036	(−0.147;0.220)
Grade (0–20)	3.505 ***	(2.920; 4.091)	1.325***	(0.786;1.863)
Agrees with assessment methodology	11,114 ***	(9.641;12.587)	4.850***	(3.400;6.300)
Perception of workload (5–25)	−4.150 ***	(−4.699;-3600)	−2.531***	(−3.169;-1.892)
Attendance	1.073	(−0.695;2.841)	0.965	(−0.261;2.191)
Agrees with teaching methodology	13.425 ***	(10.991;15.859)	8.297***	(6.279;10.315)
Score NASA (1–10)	−0.275 ***	(−0.352;-0.197)	− 0.009	(− 0.079;0.061)
Frequency of study > 0	−1.432	(−3.044;0.181)	2.217***	(1.004;4.429)
Number of students in 2015/2016 (N.x/100)	2.042 ***	(1.587;2.497)	0.848***	(0.450;1.246)
Order of preference in 2015/2016 (V1.x)	−0.288	(− 0.825;0.250)	0.537**	(0.143;0.931)

*N* = 482 surveys (out of 539 surveys)

*** < 0.001; ** < 0.01

As data were aggregated per elective, for the outcome of evaluating the determinants of the expected order of preference by elective, two models were considered: one model adjusted for the preferences established in the previous year; and a second model adjusted for the variables that had previously shown a *p*-value lower than 0.05 (Table 5).

**Table 5:** Evaluation of the determinants of the expected order of preference by elective

	ß adjusted^**a**^	(95% CI)	ß adjusted^**b**^	(95% CI)
**Global Satisfaction (V2)**	−0.031	(− 0.073; 0.012)	–	
**Grade (V3)**	−0.234	(−0.452; − 0.017)	–	
**Agrees with assessment methodology (V4)**	−1.128	(−2.059;-0.196)	− 1.333 **	
**Attendance (V5)**	−1.082	(−2.299;0.136)	−1.395 *	
**Score NASA (V6)**	−0.002	(−0.059; 0.054)	–	
**Order of preference in 2015/2016** **(V1.x) (V7)**	0.817	(0.638;0.996)	0.830 ***	

*N* = 40 electives

*** < 0.001; ** < 0.01; * < 0.05

^a^ Adjusted for preferences in the previous year (V1.x)

^b^ Adjusted for the agreement with assessment methodology (V4), for students’ attendance (V5) and for the Score NASA (V6) [15, 16]

**Fig. 1:**
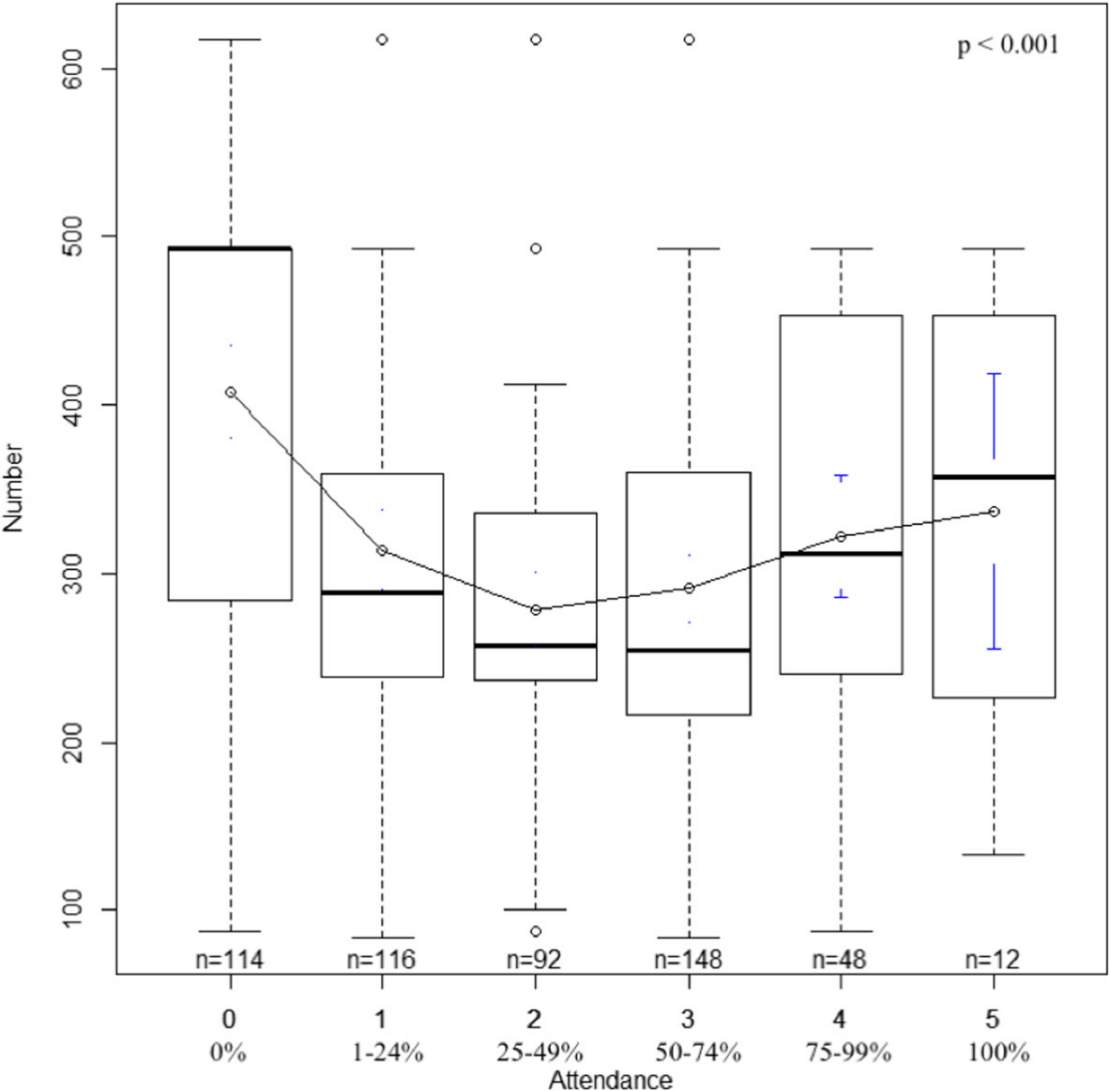
Number of students selecting electives in academic year 2015/2016 according to the classes’ attendance. Figure 1 explores the association between class attendance and the number of students who selected the elective in their personal preferences. It shows that the higher or lower the classes attendance was, the greater the number of students that want to enroll in the elective (*p* < 0.001)

**Fig. 2:**
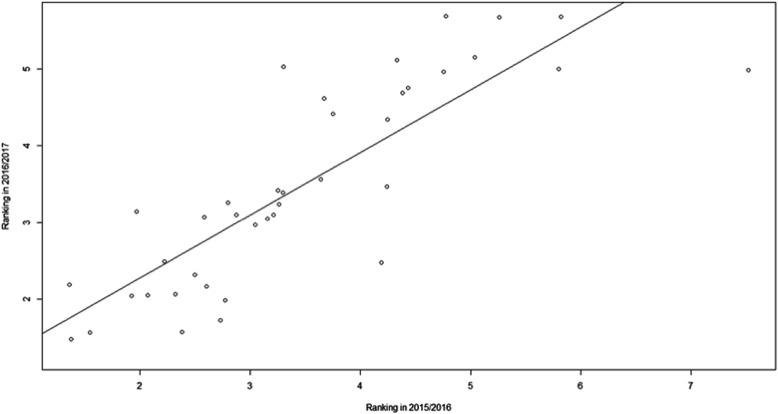
Relation between ranking of the elective in the academic years 2015/2016 and 2016/2017. Figure 2 studies the relation between students’ preferences in two consecutive years through Pearson’s correlation. The Pearson’s correlation r observed was 0.836 (95% C.I.: 0.707–0.911), meaning that there is a strong positive relationship between students’ preferences in two consecutive years

Furthermore, Fig. 1 explores the association between class attendance and the number of students who selected the elective in their personal preferences. We also studied the relation between students’ preferences in two consecutive years (Fig. 2) through Pearson’s correlation. We considered that a strong positive relationship existed when *r* > 0.700 [17].

Open-ended questions were not formally analyzed as these were not used with the purpose to generate new qualitative data. Instead, they were used to sustain and develop the close-ended answers [18]. Therefore, phenomenography [19] was used, allowing us to understand how students experienced electives in their medical curriculum.

Later, a seven-step iterative approach to analyze the open-ended responses [20] was performed by the team of investigators, starting by getting familiarized with the transcripts, being provided equal importance to the entire data obtained. After reading several times the answers to open-questions, we identified meaningful units by condensing the information in the transcripts. These units were compared regarding their similarities and the answers expressing similar understandings were then grouped into categories. Initially, there were six major relevant categories, further grouped into smaller categories (topics). The aim was to reduce the number of dimensions effectively without losing relevant information [21]. Sentences that represented these categories and topics were written down, capturing the essential meaning of each one. Both categories and topics were then labeled and associated to a theme, in order to express the core meaning of the category and topic. The identified themes were discussed within the team of investigators in several rounds, until an agreement concerning the final set was reached, in a process described as “negotiated consensus” [22]. Empirical observations were made on the quality of the assignments submitted by students.

Subsequently, the categories and topics were compared and triangulation was performed by the team of investigators in order to increase the trustworthiness of the phenomenography approach [23, 24]. At least one category and comprised topic were assigned to each identified meaning unit obtained from the open-ended answers, and student responses after triangulation were tabulated as showed in Table 6, being added some transcripts that illustrate the significance of the topic.

**Table 6:** Topics mentioned in the open-ended questions, its proportion and examples of answers obtained

Category	Topic	Topic Name	Occurrence	Most relevant answers
**Assessment**	1	Adequate Assessment	3 (3.3%)	
	2	Inadequate Assessment	25 (27.5%)	“The evaluation is subjective, it would be important to understand the criteria and objectives needed to accomplish to obtain the highest grade.” (id 24)
**Organization of the Elective**	3	Typology of classes Adequate	3 (3.3%)	“Adaptation of classes to the learning needs.” (id 21)
	4	Typology of classes Inadequate	11 (12.1%)	“Our self-reflexion shouldn’t be criticized.” (id 94) “More practice is needed.” (id 109) “There could have been group work to further develop topics applicable to Medicine.” (id 124)
	5	Weak organization	11 (12.1%)	“The only problem to point was the fact that in the first classes much time was spent recalling basic concepts. I was expecting more innovation and less repetition.” (id 94)
**Contents**	6	Background knowledge insufficient	5 (5.5%)	“The elective approaches subjects very complex and of hard comprehension for those who have less base knowledge of the theme.” (id 68)
	7	Useful/Relevant	8 (8.8%)	“Extreme relevance of the subjects approached.” (id 21) “With crucial components, this is an elective that should be part of the medical curriculum as core curricular unit.” (id 194)
	8	Innovative	22 (24.2%)	“Approaches one perspective less studied of Medicine.” (id 47) “Acquirement of scientific knowledge little addressed in the course.” (id 80) “The elective includes subjects very interesting that aren’t approached in the core unit of Morfophysiology of the Nervous System.” (id 114) “Allows for unique experiences and competences that wouldn’t otherwise be acquired during the course.” (id 181)
	9	Interesting	25 (27.5%)	“Lots of classes with people from different fields of knowledge, approaching a lot of topics and allowing for students to focus on special areas of interest.” (id 223)
	10	Low interest	4 (4.4%)	
	11	Excess of contents/ Workload inadequate	12 (13.2%)	“The contents are a little bit complex. I think that approaching fewer topics and with more depth a set of essential ones would be worthy and beneficial.” (id 227)
**Working method of the Elective**	12	Involvement of the teachers	4 (4.4%)	“One of the strengths is the involvement of the teachers and the interest of the contents teached.” (id 216)
	13	Involvement of the students	9 (9.9%)	“The typology of classes allowed a lot of discussion between the teachers and the students.” (id 69) “Very enriching personally.” (id 163) “I loved this elective. It made me learn much more in practical terms than any other core curricular unit, besides making me feel realized.”(id 227)
	14	Soft Skills/Transversal Competences	11 (12.1%)	“It is an unique experience and a very educational one for our future.” (id 114) “This elective was very important for my personal and academic path.” (id 115) “Very good elective, the levels of critic reflexion are essential for any doctor.” (id 124)
	15	Allocation Process	3 (3.3%)	“Threat: low diversity of electives.” (id 83) “Allow students to repeat this elective when there aren’t enough openings.” (id 92)

## Results

### Quantitative analysis of the questionnaires

From a total of 1231 registration in electives, we obtained 538 inquiries (43.7%). The participants were 229 students from 3^rd^ and 4^th^ year in academic year 2016/2017, mainly females [167 (72.9%)], 92.2% were younger than 25 years among a population ranging from 18 to 34 years-old.

### Determinants of students’ global satisfaction with electives

Regarding the **number of students enrolling an elective**, we found a negative correlation between the number of students allocated to an elective and age of the enrolled students. We also observed a positive association with the class attendance, showing that, as the number of students enrolling in an elective increased, so did the class attendance to that elective.

The **order of preference established by students** was positively associated with both age and classes attendance and negatively associated with the agreement with both assessment and teaching methodologies, meaning that the less desired electives had higher classes attendance and older students. Contrarily, the more students agreed with the assessment and teaching methodologies, the more they preferred the elective (Table 3).

**Students’ global satisfaction with electives** (ß crude) was positively associated with 1) *the grade* the student achieved in the elective, 2) *the agreement with assessment methodologies employed, 3) the agreement with teaching methodology* and 4) with *the number of students* assigned to the elective. Nevertheless, neither *gender, age, classes attendance, requirement for continuous work* nor the *ranking of the allocated elective in the list of preferences* were significant determinants of students’ global satisfaction with electives. Furthermore, there was a negative association with *the perception of the workload demanded*, and with *the estimated workload* of the elective (Table 4).

However, after adjusting for all the variables (ß adjusted), we verified that both *the ranking of the allocated elective in the list of preferences* and *the frequency of study being more than zero hours per day*, increased students’ global satisfaction while the negative effect of *the estimated workload* on electives’ choice was attenuated. (Table 4).

**Fig. 3:**
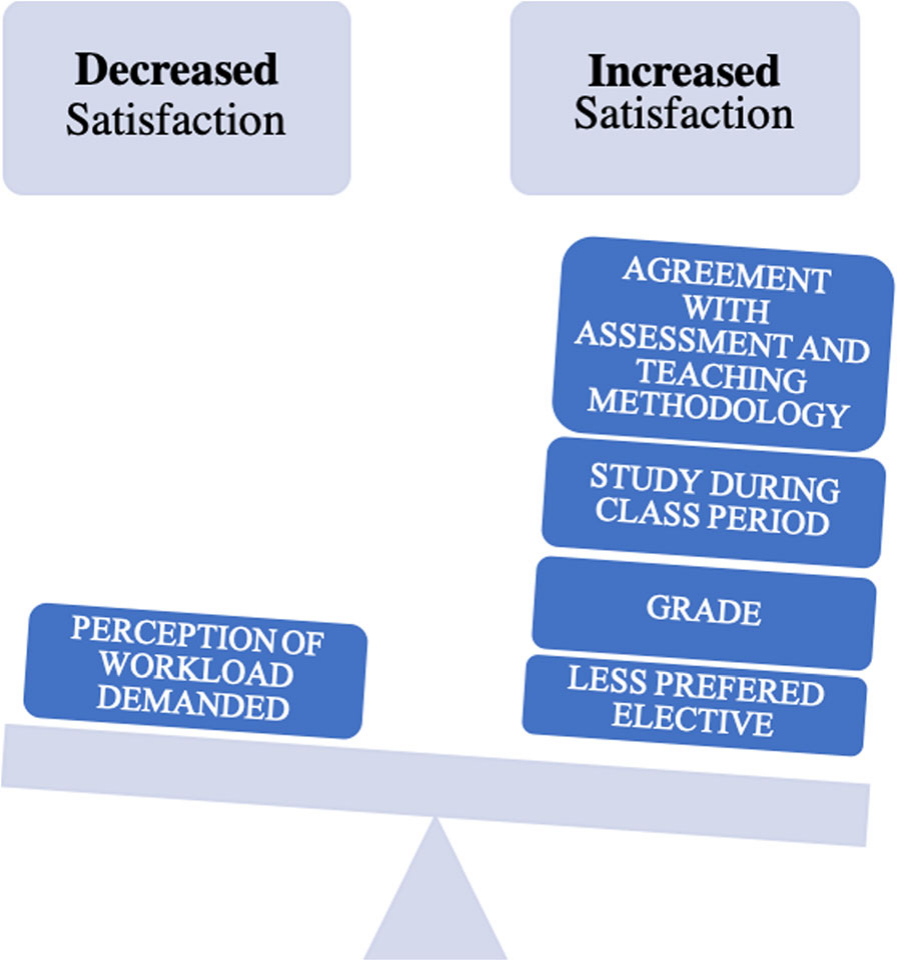
Relation of the determinants of students’ global satisfaction with electives. Figure 3 schematically shows the determinants that increase and decrease students’ global satisfation with electives

Overall the analysis of the questionnaires demonstrates that: 1) For each position that the elective decreased in students order of preferences, the global satisfaction increased 0.537 points; 2) For each additional standard deviation above the average grade of the elective, their global satisfaction increased 1.325 points; 3) If students had to study continuously during the class period (period of exams not included), the global satisfaction increased 2.217 points; 4) For each additional standard deviation in the perception of workload demanded, the global satisfaction of students decreased 2.531 points; 5) If students agreed with the assessment methodologies employed in an elective, their global satisfaction increased in 4.850 points, compared with students who do not agree and 6) If students agreed with the teaching methodologies of an elective, their global satisfaction increased in 8.297 points, compared with students who do not agree (Table 4) (Fig. 3).

Interestingly, we fount a U-shape relationship between students’ preferences and the attendance demanded by the elective, meaning that the higher or the lower the classes attendance was, the higher was the number of students that wanted to enrol in that elective (*p* < 0.001) (Fig. 1).

### Qualitative analysis of the questionnaires

Regarding the content of the open-ended answers, the free text was typically short, ranging from single words to shortlists or simple sentences. Table 6 presents representative sentences of each category, as well as the proportion of open-ended answers in which the topic occurred.

Open-ended answers provided the opportunity to enlighten about additional features not mentioned in close-ended questions, such as *the innovative* (24.2%) and *interesting* (27.5%) character of the electives, and their *relevant content* (8.8%), perceived by students. Determinants such as *the adequacy of the previous acquaintances* (5.5%), *the involvement of both teachers* (4.4%) *and students* (9.9%), as well as *the acquisition of transversal competences* (12.1%) were also acknowledged by students. The statement “Allows for unique experiences and competencies that would not have been otherwise acquired during the course” (id 181) was one of the statements that supported the innovative character of the electives offered while “With crucial components, this is an elective that should be part of the medical curriculum as core curricular unit.” (id 194) and “I loved this elective. It made me learn much more in practical terms than any other core curricular unit, besides making me feel realized.” (id 227) reinforced their relevance for the medical curriculum.

However, some answers mentioned in open-ended questions had already been evidenced by the close-ended topics. For instance, topic 11 (*Excess of contents/Workload inadequate*) overlapped simultaneously with the *Perception of the workload demanded* and the *estimated workload*, both already included in the close-ended questions of the survey. Another example of overlapped content was topic 2 (*Inadequate Assessment*), which was the one which registered the highest occurrence (27.5%) and, even though it was already assessed in the survey through questioning about the *Agreement with assessment methodologies employed*, the open-ended answer provided students the opportunity to further enlighten on the reason for their disagreement. In this case, the subjective assessment and the absence of full insight into the assessment criteria were some of the mentioned reasons that made 27.5% of students declare that the elective assessment was inadequate. The same occurred with topic 4 (*Typology of classes inadequate*), which overlapped the *disagreement with teaching methodology*, having 12.1% of the students identifying their reasons for disagreeing and mentioning opportunities for improvement.

### Determinants (predictors) of the future order of preference

We elucidated the variables that were able to predict the order of preferences defined by students for the following year, namely: 1) *the agreement with assessment methodologies employed*, 2) *classes attendance* and 3) the *ranking of the allocated elective established in the previous year*. This means that students prefer electives with which assessment methodology they agree, followed by the electives in which students have to attend classes and which students have already identified as being the most requested in previous years (Table 5). These topics explain 78.2% (R-square) of the total variance in the ranking established by students when choosing their preferred electives [25]. Expectedly, there is a significant correlation between the order of preferences established by students in a year and in the following year (r = 0.836 (95% C.I.: 0.707–0.911)) (Fig. 2).

## Discussion

When it comes to teaching-learning effectiveness, there is no single approach that best fits all students. Thus, there is a pressing need to adjust teaching/learning experience to each student [26], which justifies the existence of electives in pre-graduated curricula. Hence, we felt the need to evaluate whether students were satisfied with the variety and the quality of the electives offered by FMUP.

Lately, academic institutions have devoted an increasing amount of time and effort in developing tools that collect students feedback about their educational satisfaction. This feedback is twofold providing both the opportunity for students to express satisfaction with their educative experience and the possibility for the organization to use and process this information for improving the teaching/learning experience [27].

The existing literature states that the higher the satisfaction of students with their educative experience, the higher their learning engagement and, subsequently, the greater the ability to report an improvement in learning effectiveness [12]. Therefore, student satisfaction has become an important issue for universities and their management [27]. In order to fully achieve electives’ aims and educational potential, it is of meaningful significance to clarify the determinants of satisfaction with these selected components.

In this study, through quantitative assessment, we were able to demonstrate that the major determinant of students’ global satisfaction was the *Agreement with teaching methodology*, followed by the *Agreement with assessment methodologies employed*, the *Perception of the workload demanded* and the *Requirement for continuous work* throughout the semester. These results are similar even after standardization of the coefficients.

These findings are in accordance with the already reported teaching and learning interference on students’ satisfaction [27], justifying our need to assess their influence applied to an elective setting in the medical curriculum. Similarly, heavy workloads have been associated with a propensity to prevent students from engaging and learning an elective content [28], prompting us to assess both students’ perception of the workload demanded and students workload estimates.

Surprisingly, neither the *Grade* achieved in the electives neither the *Number of students* enrolled in the same elective were positioned as one of the main determinants, as we would expect by the causal relationship between grades and student satisfaction, and by the likelihood of large classes resulting in dissatisfaction [27, 29]. The *Ranking of the allocated elective in their list of preferences*, although influencing their global satisfaction, was unexpectedly the variable assessed that displayed the least interference.

Moreover, as there are organizational and financial restrictions that prevent universities from providing unlimited placements for each elective, developing a method that would allow predicting which electives will students select in the following years, is of extreme utility. Therefore, another important result of the present study was to help foreseeing the behavior of students when selecting their electives’ preferences. We concluded that students tended to choose electives whose assessment methodology they agreed with [*Agreement with assessment methodologies employed*], secondly, the electives in which students were expected to have higher attendance to classes during the semester [*Classes attendance*] and, lastly, the electives that had been already in their personal preference list, but which they had not been assigned previously [*Ranking of the allocated elective established in the previous year*]. In this context, we demonstrated a strong positive relationship between the order of preferences in two consecutive years, evidence that could additionally support us to predict what students’ preferences will be.

Possible weaknesses of this quantitative analysis derive from the distinct environment in which electives are managed at FMUP. For example, regarding the inference that the less desired electives had higher classes attendance and older students, one should consider that older students are mainly student-workers, that might have other demands besides medical classes. Moreover, concerning the relations identified with the *Number of students*, caution should be taken when interpreting these data, as we should take into account that this variable depends on the number of places available for each elective, which are determined by the coordinator of the elective, not depending on students’ selection options. Regarding our result that states that the global satisfaction increases 0.537 points as the electives are less prefered, we should consider a bias introduced by the possibility that the lower the expectations for the elective are, the higher the likelihood of the student to be positively surprised. Lastly, concerning the relationship between the attendance demanded by an elective and the students placing the same elective in their preference list, we should stress out that in the particular case of FMUP, these results might be twisted by an elective that simultaneously has the highest number of placements available and one of the highest demands on class attendance.

We complemented our quantitative analysis with a qualitative study of the open-ended question as we expected it would aid in deepening our understanding of students’ perceptions and in the generation of additional thoughts, to be used for the improvement of electives. We used phenomenography as it is recognized as a method that identifies the “*different ways in which people experience, conceptualise, perceive and understand various aspects*” of the world [30], exploring the variety that stands in how different people conceive the same phenomena [31]. This qualitative approach, which focuses the research on the variation among the conceptions of the medical students [32], has been proven to be viable in medical education research [20]. A bottom-up approach, through cluster analysis, was used in order to identify relevant categories or properties from textual data itself, instead of relying on predefined dictionaries which organize certain words, parts of speech or other textual properties into specific categories, as happens in top-down approach [21].

With the answers to the open-ended question, students were no longer confronted with a set of expected answers, having the opportunity to further elaborate on their close-answers, and to convey their opinions regardless of what the team of investigators perceptions and beliefs of what a complete range of answer options should be. Besides, students might intend to emphasize a feature that is of greater importance to them or to further elaborate on the answers they had provided in the close-ended questions [21]. Therefore, results drawn by this qualitative analysis allowed us to identify the reasons that lead students to choose one particular option in close-ended questions and provided further insight on students’ global satisfaction with electives, that might now be pursued.

Concerning the methodology used, phenomenography provided a way to improve the understanding of the data obtained by the close-ended answers [20]. Three criteria for evaluating phenomenography research outcome validity were met, as each category was distinctive, the categories tabulated were logically related, and the variation is represented by as few categories as possible [31].

A more objective approach for determining the adequate number of topics addressed by the open-ended questions than the bottom-up approach used for the analytical analysis of the free text generated might be desirable. However, the coherence of the derived topics from human ratings does not necessarily line up with the standard quantitative measures of model fit based on the likelihood that help to determine the adequate number of topics [13], which justified the absence of the analytical validation in the present study.

Representativeness was pursued through triangulation [24], which represents an important limitation of the analysis of the open-ended answers. Indeed, cannot ascertain if and to what extent the results allow to extrapolate conclusions to the whole sample. Nevertheless, even assuming that the representativeness was granted, it remained impossible to estimate the prevalence of a particular topic and to generalize it to all students [21]. The underlying reasons range from the possibility of the topic have been forgotten to being the most obvious at the moment of answering the survey. Hence, the contribution obtained from this subset of participants is still of great value, even if not representing the entire sample of students [18].

The reference of the innovative, interesting and relevant character of electives as well as the ability to acquire transversal competences, show one of the advantages of bottom-up data analysis, which is the provision of hints about topics that are important to respondents but were not included in the close-ended questions of the survey. Moreover, even in the case of overlap, the derived topics reassured that all relevant issues had been covered up, upholding the answers to closed questions, as well as providing further information beyond the close-ended questions [18, 21].

One limitation of this qualitative analysis is not assessing the relationship between respondents’ characteristics – gender and age – and the likelihood to answer open-ended questions, as well as the lack of testing on whether there is a relationship between global satisfaction assessed in close-ended questions and the likelihood to answer the open-ended question. Therefore, further development of the present work might be the evaluation of the relationship between answers patterns in open- and close-ended questions, allowing for the identification of which students make use of the open-ended questions and elaborating on how free texts are related to respondents’ characteristics. Moreover, pursuing a correlation analysis and developing a topic-level study should be helpful to identify topics that potentially distinguish between respondents which are more or less satisfied with electives [21], identifying positive and negative correlations between the occurrence of specific topics and students’ global satisfaction with electives, similarly to what we have already performed for close-ended questions.

Additionally, although studying the interference of electives in future career was not a goal of the present study, it would be interesting to follow the career choices of these students in order to understand the long-term contributions of electives experience to students’ future professional activities and choices, and maybe relate with different levels of satisfaction achieved throughout the undergraduate curriculum.

## Conclusions

Organization of electives in the medical curriculum should be done through a student-centered approach, in order to maximize educational, personal and professional development [3], thus, students feedback is crucial. Schools should have a role in facilitating elective choice for students, by encouraging them to be realistic about their aims, by tailoring electives to students expectations, and ensuring the optimization for high-quality elective placements [3].

Electives allow for a more in depth development of the syllabus, contributing to the diversity of experiences and scientific knowledge, while simultaneously instilling the responsibility on the learner to build its own educational path. This has been supported by some students’ statements describing electives as being innovative, interesting and offering relevant content, adding competencies to the core curricula and making students feel realized with their learning experience, which reinforces their relevance in medical curriculum.

In order to engage students, we should aim to enhance their satisfaction and atenuate factors that create dissatisfaction. Therefore, our work heightened electives’ potential in undergraduate medical curricula by unhiding the main determinants of students’ satisfaction with electives, making it possible to improve their features in order to meet their full potential and students’ expectations, adding to the current literature clear structural recommendations for electives design that may lead to students’ satisfaction and engagement.

The first step in creating a successful elective is to determine whether there is student interest in the topic. Then, the ideal course structure should be assessed [2]. The results of the present work determine that, in order to reach students’ highest level of satisfaction with electives, we must assure that teaching and assessment methodologies are adequate and that they meet students’ expectations. Furthermore, regarding the workload, electives should maximize the use of elective time without requiring extensive amounts of outside work for the class [2].

Additionally, our work provides predictors of students’ electives preferences, being their first choice the electives whose assessment methodology they agreed with, followed by the electives in which students were expected to have higher attendance to classes during the semester and, lastly, the electives that they had previously selected in their preferences. In fact, our work reinforces the usefulness of analyzing the records of the students’ order of preferences from previous years, to accurately foresee the needs of the following year, besides providing the foundations to identify patterns of choices from students, which will also contribute to better meet their needs and expectations.

Summing up, the present study allowed us to identify the most important determinants on students’ choices and satisfaction with electives, some of which allow us to foresee which electives will be selected in the future, which had not been done yet. It also distinguishes the features that make students prefer one specific elective over another, providing the foundation for future electives and curriculum design, at the same time that reveals opportunities to improve already existing practices with electives. Furthermore, it might even provide opportunities to improve current medical core curricula, namely by shifting current assessment methodologies and teaching and learning methodologies towards the ones most preferred by medical students.

Moreover, our data strongly reinforces the need to closely monitor the implementation of electives in medical curricula as crucial dynamic elements of the medical curricula, opposing the tendency of maintaining the electives untouched in the curricula of medical schools, compromising their potential [3].

Finally, we point out potential opportunities for improving the organization of the medical curricular reform ongoing at FMUP. These improvements includes, among others, the introduction of electives as a crucial dynamic part of the curricula, that is, to develop a comprehensive and adaptive model of medical education so that FMUP medical students become referenced professionals, equipped with the enhanced skills to better improve people’s health, through excellence in clinical practice, research, innovation and leadership.

## Supplementary information

**Additional file 1.** Assessment of the quality of Electives in the 1st/2nd semester. The Additional File 1 represents the written evaluation survey handed once to students who attended electives when they were in the 2nd and 3rd years, in the academic year of 2015/2016.

## Data Availability

The datasets used and/or analysed during the current study are available from the corresponding author on reasonable request.

## Abbreviation

FMUP: Faculty of Medicine of University of Porto
