# Supplementary material for: Electives in the medical curriculum – an opportunity to achieve students’ satisfaction?
Source: BMC Med Educ. 2020 Nov 23;20:449. doi: 10.1186/s12909-020-02269-0 (PMC7681969; doi:10.1186/s12909-020-02269-0)
Supplement: Supplementary file 1 — Additional file 1. Assessment of the quality of Electives in the 1st/2nd semester. The Additional File 1 represents the written evaluation survey handed once to students who attended electives when they were in the 2nd and 3rd years, in the academic year of 2015/2016. [file 12909_2020_2269_MOESM1_ESM.pdf]

U.PORTO
FMUP FACULDADE DE MEDICINA
UNIVERSIDADE DO PORTO

(Do Not Fulfil) (Do Not Fulfil)

Assessment of the quality of Electives in the 1st/2nd semester

Semester:

Write the elective that you have attended in the 1st/2nd **semester**:

1.1. Age: 1.2. Gender: O Male O Female 1.3. Write the grade obtained in the elective:

Workload
When you answer think in THE elective as a whole instead of a topic, class or subject in particular. The questions are related with the workload in the elective during the previous curricular year. The items are punctuated in a scale of 1 to 5 where 1 means "**Strongly Disagree**" and 5 "**Strongly Agree**".

	Strongly Disagree	Disagree	Neither Agree/ Nor Disagree	Agree	Strongly Agree
2.1. The workload of the elective is too high.	O 1	O 2	O 3	O 4	O 5
2.2. It seems that the programmatic content tends to comprise too many topics.	O 1	O 2	O 3	O 4	O 5
2.3. During classes I generally have enough time to understand the contents.	O 1	O 2	O 3	O 4	O 5
2.4. There is high burden on the student in this elective.	O 1	O 2	O 3	O 4	O 5
2.5. The overload of work in this elective doesn't allow me the exhaustive comprehension of all concepts.	O 1	O 2	O 3	O 4	O 5

During the class period
3.1. Point the % of classes that you have attended in this elective:
O 0% O [1 a 24%] O [25 a 49%] O [50 a 74%] O [75 a 99%] O 100%

3.2. Point with which frequency you have studied for this elective during the class period:
O Never O <=1/month O 2-3/month O 1/week O 2/week O 3-4/week O 5/week O 6-7/week

3.2.1. 1 In the days that you have studied, point the average number of hours that you have studied per day for this elective:

, Hours/day

4. The elective has assessment during exams period: O No O Yes

4.1. **If no,** go forward to **question 5.**
If yes, point the number of days that you have studied for this elective during exams period: Days

4.1.1. In the days that you have studied, point the average number of hours that you have studied per day for this elective: , Hours/day

Students' perception regarding the quality of the elective
When you answer think in THE elective as a whole instead of a topic, class or subject in particular. The questions are related with the workload in the elective during the previous curricular year. The items are punctuated in a scale of 1 to 5 where 1 means "**Strongly Disagree**" and 5 "**Strongly Agree**".

	Strongly Disagree	Disagree	Neither Agree/ Nor Disagree	Agree	Strongly Agree
5.1. The elective was what I expected.	O 1	O 2	O 3	O 4	O 5
5.2. The bibliography/material provided for the elective was useful.	O 1	O 2	O 3	O 4	O 5
5.3. In this elective I have learned a lot.	O 1	O 2	O 3	O 4	O 5
5.4. In my opinion this elective was pleasant and useful.	O 1	O 2	O 3	O 4	O 5
5.5. The teacher(s) was(were) well succeeded in encouraging students to think independently about the theme of this elective.	O 1	O 2	O 3	O 4	O 5
5.6. The assessment of this elective was appropriate.	O 1	O 2	O 3	O 4	O 5
5.7. I would recommend this elective to my colleagues.	O 1	O 2	O 3	O 4	O 5
5.8. If I could choose again, I would choose again this elective.	O 1	O 2	O 3	O 4	O 5

5.9. Globally I am satisfied/unsatisfied with the quality of this elective.
O Very Satisfied O Satisfied O Neither Satisfied/Nor Unsatisfied O Unsatisfied O Very Unsatisfied

Assessment components

6.1. Do you agree with the current assessment components in this elective?

- ○ No **If not**, go forward to **question 6.3.**
- ○ Yes **If yes**, go forward to **question 6.2.**

6.2. 2 Do you agree with the weight attributed to each of the components in this elective?

- ○ No **If not**, go forward to **question 6.3.**
- ○ Yes **If yes**, go forward to **question 7.**

6.3. Point which assessment components of the elective and the corresponding weight that should be assigned to each component (making a total of 100%). If you answered No in question 6.2 point the weight that should be assigned in the assessment components that are included in the elective (making a total of 100%).

Final Exam	○ No ○ Yes	**If yes**, point the weight:		%
Attendance	○ No ○ Yes	**If yes**, point the weight:		%
Test (frequency)	○ No ○ Yes	**If yes**, point the weight:		%
Written work	○ No ○ Yes	**If yes**, point the weight:		%
Oral test	○ No ○ Yes	**If yes**, point the weight:		%
Laboratorial work	○ No ○ Yes	**If yes**, point the weight:		%

Classes Typology

7.1. Do you agree with the current classes typologies in this elective?

- ○ No **If not**, go forward to **question 7.2.**
- ○ Yes **If yes**, go forward to **question 8.**

7.2. Point which should be the classes typologies in this elective:

Theoretical	○ No ○ Yes	Practical	○ No ○ Yes	Other:
Theoretical-Practical	○ No ○ Yes	Seminar	○ No ○ Yes	

8. The following scale assesses the amount of work invested in 6 dimensions. For each one of the dimensions point in a scale from 1 to 10, in which 1=Low and 10=High, the answer that best represents your level of effort for the completion of this elective.

Mental Effort: Which was the level of mental effort and perception demanded?

Physical Effort: Which was the level of physical effort demanded?

Temporal Effort: Which was the level of pressure imposed by the time to complete the activities?

Performance: Which was the level of success felt in the accomplishment of the aims?

Effort: Which was the level of effort that you had to spend to accomplish your results?

Frustration: Which was the level of insecurity, discouragement, irritation and stress that you felt?

Strengths / Weaknesses / Opportunities / Threats
